# Aptasensor for the Detection of *Moraxella catarrhalis* Adhesin UspA2

**DOI:** 10.3390/bioengineering10020178

**Published:** 2023-01-31

**Authors:** Maria G. Sande, Débora Ferreira, Joana L. Rodrigues, Luís D. R. Melo, Athanasios Saragliadis, Dirk Linke, Felismina T. C. Moreira, Maria Goreti F. Sales, Ligia R. Rodrigues

**Affiliations:** 1 CEB—Centre of Biological Engineering, Universidade do Minho, Campus de Gualtar, 4710-057 Braga, Portugal; 2 LABBELS—Associate Laboratory, 4710-057 Braga, Portugal; 3 Section for Genetics and Evolutionary Biology, Department of Biosciences, University of Oslo, 0316 Oslo, Norway; 4 BioMark-CINTESIS/ISEP, School of Engineering, Polytechnic Institute of Porto, 4219-015 Porto, Portugal

**Keywords:** adhesins, outer membrane proteins (OMPs), ubiquitous surface protein A2 (UspA2), point-of-care (PoC), biosensor, electrochemical, cell-SELEX, aptamers, detection

## Abstract

Innovative point-of-care (PoC) diagnostic platforms are desirable to surpass the deficiencies of conventional laboratory diagnostic methods for bacterial infections and to tackle the growing antimicrobial resistance crisis. In this study, a workflow was implemented, comprising the identification of new aptamers with high affinity for the ubiquitous surface protein A2 (UspA2) of the bacterial pathogen *Moraxella catarrhalis* and the development of an electrochemical biosensor functionalized with the best-performing aptamer as a bioreceptor to detect UspA2. After cell-systematic evolution of ligands by exponential enrichment (cell-SELEX) was performed, next-generation sequencing was used to sequence the final aptamer pool. The most frequent aptamer sequences were further evaluated using bioinformatic tools. The two most promising aptamer candidates, Apt1 and Apt1_RC (Apt1 reverse complement), had *K_d_* values of 214.4 and 3.4 nM, respectively. Finally, a simple and label-free electrochemical biosensor was functionalized with Apt1_RC. The aptasensor surface modifications were confirmed by impedance spectroscopy and cyclic voltammetry. The ability to detect UspA2 was evaluated by square wave voltammetry, exhibiting a linear detection range of 4.0 × 10^4^–7.0 × 10^7^ CFU mL^−1^, a square correlation coefficient superior to 0.99 and a limit of detection of 4.0 × 10^4^ CFU mL^−1^ at pH 5.0. The workflow described has the potential to be part of a sensitive PoC diagnostic platform to detect and quantify *M. catarrhalis* from biological samples.

## 1. Introduction

*Moraxella catarrhalis* is a diplococcal, gram-negative bacteria and an exclusive human pathogen that has been isolated from the nasopharynx, throat, ear effusions and sinus aspirates of patients [1]. *M. catarrhalis* is a frequent etiological agent of otitis media in children and accounts for 10% of bacterial exacerbations in adults with chronic obstructive pulmonary disease [2], being traditionally diagnosed by culture combined with gram staining and morphological identification [3]. However, cultivation is difficult, insensitive and time-consuming due to its fastidious nature. Moreover, owing to its morphological and phenotypic similarity to the *Neisseria* species [1], additional lab tests are typically required to distinguish between them [3]. Nucleic acid amplification methods and matrix-assisted laser desorption/ionization time-of-flight mass spectrometry (MALDI-TOF-MS) have been used for this purpose [4,5,6,7,8]. Although these latter methods possess superior sensitivity and are quickly accomplished, they are also expensive, require specialized technicians and can only be performed in a well-equipped laboratory. These limitations are part of a broader dilemma with traditional diagnostic methods for infections, making them inaccessible both physically and financially to most patients who develop infectious diseases who, incidentally, live in underdeveloped parts of the world. To help mitigate this problem of lack of access, in vitro diagnostic tools, also referred to as point of care (PoC) devices or methods, are being developed, and lateral flow assays for some infections are commercially available [9]. PoC platforms must preferably be self-contained, inexpensive, simple to use and clinically proven to reliably fulfill their purpose of making diagnostics accessible to almost anyone, anywhere [10].

In general, to design a working PoC biosensor, careful consideration needs to be given to the selection of (i) the principle of detection, (ii) the analyte, which is the target derived from the pathogen (it can also be the pathogen itself) and (iii) the biorecognition element or probe, which recognizes and binds to the analyte sensitively and selectively. With regards to the choice of the sensing principle, electrochemical biosensors are one of the most widely investigated. This is due to their ease of assembly, low cost, potential for miniaturization [11] and detection capabilities over a range of analytes for the purpose of food and water safety, medical diagnostics, environmental monitoring and other applications [12]. In this type of biosensor, an electrode is used as a solid support for the immobilization of biomolecules and electron movement [13]. These types of biosensors have been widely and successfully employed for the detection of various pathogenic moieties, including biosensors functionalized with aptamers [14,15,16,17,18].

To assemble an electrochemical biosensor towards the detection of *M. catarrhalis,* the bacterial outer membrane proteins (OMPs) are possible analyte candidates due to their location on the surface, ubiquity and immunogenicity. The non-fimbrial ubiquitous surface protein A2 (UspA2) is a prominent, high-molecular-weight OMP that is abundantly expressed on the surface of *M. catarrhalis* giving the bacteria a fuzzy appearance [19]. Structurally, UspA2 is a multifunctional trimeric autotransporter adhesin (TAA) comprising a “lollipop”-like head, coiled-coil stalk and membrane anchor regions [20,21]. UspA2 is an important virulence factor which mediates adhesion to various human cell receptors and proteins, contributes to bacterial serum resistance [22] and exhibits auto-aggregation [23]. In the past, several efforts have been made to develop detection/diagnosis methods (e.g., antigen-antibody detection [24,25], reverse-transcription PCR [24], mass spectrometry [7]) by exploiting these particular analytes (OMPs, UspA2). But none of these have been developed to a PoC product so far. Therefore, the development of an electrochemical PoC platform which can detect UspA2 from samples for the diagnosis of *M. catarrhalis* infections is timely and relevant.

A good biorecognition element must bind specifically to the analyte and with sufficiently high affinity so that the biosensor can accurately and sensitively detect the analyte at clinically relevant concentrations [9]. Aptamers are promising candidates for this purpose, being isolated in vitro from randomized libraries by a technique called systematic evolution of ligands by exponential enrichment (SELEX) [26]. They have advantages over antibodies, including superior stability, versatility, smaller size, reduced immunogenicity and lower production costs [27]. When the targets during SELEX are whole live cells, it is termed cell-SELEX. Cell-SELEX has the advantage that aptamers can be selected against targets in their natural form [28]. Several examples of aptamers selected by bacterial cell-SELEX have been reported, and they are very popular probes in biosensors [16,27,29,30].

In this study, the development and evaluation of a simple electrochemical biosensor to detect UspA2 of *M. catarrhalis* using aptamers as a biorecognition element are reported. Aptamers selectively binding with high affinity to UspA2 expressed in engineered *Escherichia coli* were identified using cell-SELEX. The most promising aptamer was immobilized on a gold-screen-printed electrode (Au-SPE) to successfully detect UspA2 with high sensitivity. The goal of this study is to contribute to the development of PoC solutions for the diagnosis of *M. catarrhalis* infections. Towards this goal, the approach and techniques used in this study can serve as a proof-of-concept for the development of PoC biosensors able to detect other clinically relevant bacteria.

## 2. Materials and Methods

### 2.1. Bacterial Strains and Plasmids

*E. coli* Top10 competent cells were used for plasmid construction and propagation. The *uspA2* gene of *M. catarrhalis* Bc5 (GenBank number: AGH27427.1) was amplified from its genomic DNA by colony-PCR using Phusion High-Fidelity DNA Polymerase (New England Biolabs (NEB), Ipswich, MA, USA). The primers (Table S1) were designed with overhangs complementary to the pASK-IBA2 plasmid (IBA BioTAGnology, Göttingen, Germany) for subsequent annealing during AQUA (advanced quick assembly) cloning [31]. Plasmid pASK-IBA2 was linearized by PCR, also with Phusion High-Fidelity DNA Polymerase and the respective primers. Upon PCR cleanup, the plasmid template was digested with *Dpn*I (NEB) restriction endonuclease for 30 min at 37 °C in Cutsmart buffer (NEB). Thereafter, *Dpn*I was inactivated by incubation at 80 °C for 10 min. Figure S1 shows a simple scheme illustrating the steps involved in AQUA. Successful cloning was verified by colony PCR and sequencing (Eurofins Genomics, Ebersberg, Germany). Finally, the resulting plasmid pASK-IBA2_UspA2 was propagated in *E. coli* BL21 (DE3) (NZYTech, Lisbon, Portugal) for protein expression and for use in subsequent experiments. This strain will simply be referred as *E. coli* UspA2 from now on. *E. coli* BL21 (DE3) carrying the empty plasmid pASK-IBA2 used as a negative control in experiments will be referred to as *E. coli* IBA.

### 2.2. Sedimentation Assay

*E. coli* UspA2 and *E. coli* IBA cultures grew in 50 mL Lysogeny Broth (LB, NZYTech) in conical flasks supplemented with ampicillin (100 µg mL^−1^ final concentration, NZYTech), incubated at 37 °C, 200 rpm. When an optical density at 600 nm (OD_600nm_) of 0.7 was attained after approximately 3 h, a sample of 5 mL (corresponding to time 0) was transferred to a 15 mL falcon tube. After this, expression of UspA2 was induced in the growing cultures by adding anhydrotetracycline (200 µg L^−1^, Acros, Geel, Belgium). Subsequently, 5 mL samples were taken every hour for 5 h. Samples were taken in duplicate. After 5 min of settling down, 250 µL samples were taken from the surface of the 5 mL sample tubes to measure OD_600nm_ to evaluate sedimentation. The OD_600nm_ was then plotted as a function of time.

### 2.3. Protein Analysis

*E. coli* UspA2 culture was prepared in 5 mL LB supplemented with ampicillin as previously described. At an OD_600nm_ of 0.7, UspA2 expression was induced with anhydrotetracycline. Samples (1 mL) were collected in 1.5 mL tubes just prior to induction time (time 0) and at intervals of 1 h after induction for 3 h. Afterwards, the samples were centrifuged (5000× *g* 10 min) and each pellet was resuspended in 1 mL of Tris–HCl buffer (50 mM, pH 7.8). Cells were lysed by sonication using a microtip probe linked to a Vibra-cell processor (Sonics, Newtown, CT, USA). The samples were kept on ice during the procedure. Short pulses of 15 s ON and 10 s OFF at 30% amplitude were performed for 3 min of active sonication. The resulting lysate was centrifuged (13,000× *g*, 10 min). The soluble fraction of each sample was decanted and the insoluble phase was collected separately after resuspending the pellets in 1 mL of Tris–HCl buffer (50 mM, pH 7.8). Both insoluble and soluble samples were analyzed to verify UspA2 expression. Protein concentration in the soluble and insoluble fractions was measured using the Pierce™ Coomassie (Bradford, UK) Protein Assay Kit in accordance with manufacturer instructions.

The expression of UspA2 was evaluated by sodium dodecyl sulfate polyacrylamide gel electrophoresis (SDS–PAGE) (8% stacking gel and 12% running gel). Samples (10 µg protein) were mixed with 2× Laemmli loading Buffer (65.8 mM Tris–HCl pH 6.8 (Fisher Scientific, Loughborough, UK), 2.1% SDS (Fisher-Scientific), 26.3% glycerol (JMGS, Odivelas, Portugal), 0.01% bromophenol blue (Sigma-Aldrich, Steinheim, Germany) and 5% β-mercaptoethanol (AppliChem, Darmstadt, Germany)) and were denatured at 95 °C for 5 min. The protein marker used was the Color Prestained Protein Standard, broad range (11-245 kDa) (NEB). After electrophoresis, the gel was stained with Coomassie Blue R-250 (AppliChem) for 15 min and de-stained with distilled water.

### 2.4. ssDNA Library, Aptamer Sequences and Primers

A randomized ssDNA library comprising 50 nucleotides (nt) flanked by constant primer binding regions (Table S2) was used to start the cell-SELEX assays. After each cell-SELEX cycle, a PCR amplification step was performed using a forward and a reverse primer complementary to the library (Table S2). The library and primers were purified by high performance liquid chromatography (HPLC, Invitrogen, Waltham, MA, USA). For characterization experiments, the candidate aptamers Apt1 and its reverse complement (RC) sequence (Apt1_RC) were labeled with fluorescein (FAM) (Metabion, Steinkirchen, Germany) (Table S2). For immobilization on Au-SPEs during assembly of the biosensor, Apt1_RC was modified with thiol (Metabion) and purified by HPLC.

### 2.5. In Vitro Selection of Aptamers by Cell-SELEX

An *E. coli* UspA2 culture was prepared in 5 mL LB and supplemented with ampicillin as previously described. When an OD_600nm_ of 0.7 was reached after approximately 3 h, expression of UspA2 was induced by adding anhydrotetracycline and the culture was grown for an additional 1.5 h. Next, the cells from 500 µL of the *E. coli* UspA2 culture were harvested, washed in phosphate buffer saline (PBS 1x, pH 7.4 (137 mM NaCl (NZYTech), 2.7 mM KCl (ChemLab, Zedelgem, Belgium), 8 mM Na_2_HPO_4_ (ChemLab) and 2 mM KH_2_PO_4_ (Panreac, Barcelona, Spain)) and resuspended in 600 µL of selection buffer (SB: PBS 1x, pH 7.4 supplemented with 2 mM KH_2_PO_4_ (Panreac) and 1.4 mM MgCl_2_·6H_2_O (VWR, Radnor, PA, USA)). During counter-selection cycles, *E. coli* IBA culture was resuspended in 100 µL SB.

Cell-SELEX cycles: A schematic illustration of the steps of the cell-SELEX cycles is shown in Figure S2. A volume of 200 µL (2 nmol) of ssDNA library was prepared in SB and heated (5 min, 95 °C) followed by cooling to room temperature. To initiate the first selection cycle, the 200 µL of library was mixed with the 600 µL of *E. coli* UspA2 and incubated for 60 min, at 37 °C and with gentle shaking. Subsequently, the supernatant was discarded and the cells recovered (during counter-selection cycles, the supernatant was saved and used for PCR-amplification of the sequences present). The cells were washed in washing buffer (PBS 1x) to eliminate any remaining unbound sequences and then suspended in 100 µL DNase-free water and heated at 95 °C, 5 min. These eluted sequences were PCR amplified. In the following cycles, the ssDNA obtained from the preceding cycle (instead of the library) was incubated with fresh *E. coli* UspA2 culture. Eight cycles of cell-SELEX were performed in total. The 5th and 7th cycles were counter-selections or negative selections. During these cycles, *E. coli* UspA2 cells were replaced by *E. coli* IBA cells corresponding to a negative control to remove any aptamer sequences recognizing surface molecules common to both *E. coli* strains. The stringency of each of the eight cycles was increased by varying certain parameters, including the time of incubation, the volume of cells used for positive selection and the number of washes after selection (refer to Table 1).

PCR amplification and preparation of ssDNA: The conditions used for PCR reactions performed after each selection cycle to amplify the eluted sequences are described here. HiFi DNA Polymerase Master Mix (VWR, Radnor, PA, USA) was used for amplification. Primers (final concentration of 0.2 μM) are presented in Table S2. The PCR conditions were: initial denaturation at 95 °C for 5 min, 13–30 cycles (Table 1) of 30 s denaturation at 95 °C, 30 s annealing at 68–72 °C and 30 s extension at 72 °C, and a final extension of 5 min at 72 °C. After each reaction, a small PCR product (85 nt) amount was analyzed in an agarose gel (3%) to confirm the presence of the dsDNA with a band of the right size. The cleanup of the PCR product was performed using a MiniElute PCR purification kit (Qiagen, Germantown, MD, USA). Finally, to separate the strands of the dsDNA PCR product, it was heated for 5 min at 95 °C and then cooled to obtain ssDNA, ready for use in the next selection cycle.

### 2.6. Sequencing and Data Analysis

The ssDNA pool from the last (8th) cell-SELEX cycle was sequenced (Stab Vida, Caparica, Portugal), with the Illumina Novaseq system (short-read process) and using 150-nt paired-end sequencing reads. The raw next-generation sequencing (NGS) data obtained was analyzed with an alignment algorithm from the Geneious software 9.1.4 (Biomatters Ltd., Auckland, New Zealand; https://www.geneious.com/ accessed on 25 July 2022). The constant primer binding regions were removed and the sequences were filtered by discarding those longer or shorter than 46–52 nt. The 10 most frequently occurring oligonucleotide sequences, and the respective reverse complement sequences, were identified and further evaluated using the Mfold web server (version 3.0, http://www.unafold.org/mfold/applications/dna-folding-form.php, accessed on 17 July 2022 and 12 December 2022) with regards to their secondary structures, thus, presenting an estimation of the optimal structure for each aptamer and their correspondent Gibbs free energy (∆G). During prediction of the secondary structures using Mfold, the conditions used were set based on the composition of the SB (137 mM Na^+^ and 1.4 mM Mg^2+^ at pH 7.4) at 37 °C. Furthermore, the alignment of these sequences and the construction of their phylogenetic tree were accomplished with the Tree Builder function in Geneious. This function allowed to estimate the tree distances and sequence relatedness using a neighbour-joining model.

### 2.7. Binding Experiments

To calculate the dissociation constants (*K_d_*) of candidate aptamers Apt1 and Apt1_RC, *E. coli* UspA2 and *E. coli* IBA cultures were prepared in 5 mL LB supplemented with ampicillin and grew at 37 °C, 200 rpm. At an OD_600nm_ of 0.7, protein expression was induced with anhydrotetracycline in the *E. coli* UspA2 culture and the cells grew for 1.5 h more. Subsequently, the *E. coli* UspA2 and *E. coli* IBA cells were harvested, washed in PBS 1× and resuspended in 5 mL SB. Microcentrifuge tubes containing increasing concentrations of FAM-labeled Apt1 or FAM-labeled Apt1_RC (0, 3, 5, 10, 20, 30, 50, 75 and 100 nM) were prepared in duplicate and mixed with *E. coli* UspA2 or *E. coli* IBA (negative control measurements) (OD_600nm_ ≈ 1) to a total volume of 250 μL. Thereafter, all tubes were incubated for 75 min at 37 °C with gentle shaking. Next, the cells from each sample were recovered, washed and resuspended in 250 μL of SB. The cell-bound FAM-labeled aptamers were eluted by heating at 95 °C for 5 min. Thereafter, the supernatant of each sample containing the eluted aptamers was recovered and transferred to a 96-well dark microtiter plate (Corning, Sigma, Darmstadt, Germany). The fluorescence intensity was measured with a Cytation 3 spectrophotometer (excitation: 492 nm, emission: 518 nm) (BioTek, Winooski, VT, USA). To determine the *K_d_* of the aptamer-cell interactions, the mean fluorescence intensity of cells (Y) was plotted against the aptamer concentration (X, nM) and the binding capacity (*B_max_*, maximum number of binding sites), according to Equation (1) using a non-interacting binding sites model. To perform the computations, GraphPad Prism 7 (San Diego, CA, USA, version 7.00 for Windows) was used.

(1) $$Y=\frac{B_{max}\;X}{\left(K_{d}+X\right)}$$

### 2.8. Electrochemical Apparatus, Electrodes and Reagents

A potentiostat/galvanostat PGSTAT320N from Metrohm Autolab (Utrecht, The Netherlands) was used for electrochemical measurements and the NOVA 1.11 software (Utrecht, Netherlands) facilitated data acquisition and processing. Au-SPEs were purchased from DropSens (Asturias, Spain) (DS-C220AT). An SPE comprises a gold working electrode with a 4 mm diameter (Figure S4A), a counter electrode of platinum and a reference electrode with silver electrical contacts. The geometric area of each electrode was 0.125 cm^2^. The working surface of an Au-SPE under a scanning electron microscope is shown in Figure S4B. The electrodes were connected to a potentiostat using a switchbox from DropSens.

To evaluate the electrical properties of the modified electrode surfaces, electrochemical impedance spectroscopy (EIS), cyclic voltammetry (CV) and square wave voltammetry (SWV) assays were performed in a redox probe solution of potassium hexacyanoferrate-III (K_3_ [Fe (CN)_6_]) 5.0 × 10^−3^ M and potassium hexacyanoferrate-II (K_4_ [Fe (CN)_6_]) 5.0 × 10^−3^ M, prepared in PBS 1× buffer, pH 7.4. K_3_ [Fe (CN)_6_] and K_4_ [Fe (CN)_6_] were obtained from Riedel-de Haen (Seelze, Germany). Electrochemical assays were created in triplicate. To perform the EIS assays, an open circuit potential was applied and a sinusoidal potential perturbation of amplitude 0.01 V and 50 data points was distributed logarithmically over a frequency range of 0.1–100,000 Hz. EIS data was fitted to a Randles’ equivalent circuit (Figure S5). Scanning potentials from −0.4 to +0.6 V, at a scan rate of 50 mV/s were used in CV assays. To perform SWV assays, a potential ranging from −0.4 to +0.6 V and at a frequency of 20 Hz, with a step height of 50 mV was used.

### 2.9. Assembly of the Aptasensor

Each Au-SPE used was first cleaned with absolute ethanol (99.5%, Panreac) and thoroughly washed with ultrapure water to remove all traces of ethanol and impurities. To assemble the biosensor, first, the Au-SPE was cleaned, followed by immobilization of the thiolated Apt1_RC on its surface. Ultrapure Mili-Q laboratory grade (conductivity < 0.1 µS/cm) was always used, and PBS 1× was prepared freshly from tablets (Amresco, Dallas, USA). The electrode active area was calculated using the Randles-Sevcik equation and found to be 0.202 cm^2^ before cleaning and 0.215 cm^2^ after cleaning.

The aptasensor was assembled in two simple steps, namely (i) immobilization of the probe on the Au-SPE, followed by (ii) blocking of non-specific binding sites. Firstly, a stock solution of thiolated Apt1_RC (1 µM) in phosphate buffer (5 mM MgCl_2_ (Riedel-de Haen), in PBS 1×, pH 7.4) was prepared. Before immobilization on the electrode, the stock solution of Apt1_RC was further diluted in phosphate buffer to a working concentration of 0.5 µM and deprotected by incubation with dithiothreitol (DTT, 0.1 M, Sigma Aldrich, Steinheim, Germany) for 30 min at room temperature followed by heating at 95 °C for 5 min. Deprotection disrupts disulphide bonds, and heating makes the aptamer flexible and interactive. The Apt1_RC (5 µL) was then immediately added to the Au working surface and incubated in a glass chamber at room temperature for 2 h. In this step, the aptamer is immobilized via thiol groups at its 5′ end. Subsequently, the Au surface was washed several times to remove all unbound Apt1_RC probes. The Au-SPE was then incubated with 6-mercapto-1-hexanol (MCH, 5 mM, TCI Chemicals, Zwijndrecht, Belgium) for 2 h at room temperature to block unoccupied sites on the Au surface [32]. This step was crucial to prevent non-specific binding of biomolecules present in a sample to the Au surface. All steps of the aptasensor assembly were monitored by CV and EIS assays to confirm the modifications created to the Au-SPE. For this purpose, 80 µL (5 mM) of the redox probe was added to an Au-SPE that had been modified, followed by recording of CVs and EIS Nyquist plots of the redox probe.

### 2.10. Evaluation of the Aptasensor in Standard Solutions

To evaluate the performance of the aptasensor to detect UspA2, calibration of the sensory surface was performed with six standard solutions of inactivated *E. coli* UspA2 (7.0 × 10^4^ to 7.0 × 10^7^ CFU mL^−1^). To prepare an inactivated stock of *E. coli* UspA2, a culture of 50 mL of *E. coli* UspA2 cells (7.0 × 10^7^ CFU mL^−1^) was prepared in LB as previously described and the cells harvested. To inactivate the cells, 10 mL of ethanol (70%) was added to the cell pellet for 5 min and then it was thoroughly removed. Finally, the inactivated *E. coli* UspA2 cells were resuspended in 50 mL of PBS 1× (pH 7.4). Serial dilutions of this stock were prepared in two different buffers with different pH (1 mM PBS 1×, pH 7.4; or 1 mM 2-(N-orpholino) ethane sulfonic acid and 4-morpholine ethane sulfonic acid (MES) monohydrate (98%, AppliChem, Darmstadt, Germany), pH 5.0).

During calibration assays, standard solutions of inactivated *E. coli* UspA2 in buffer were sequentially added (≈5 μL) onto the Au-SPE working surface of the aptasensor and incubated at 37 °C for 30 min in a humidified environment. After taking electrochemical readings, cells were removed with ultra-pure water. Each incubation was followed up with electrochemical assays performed as usual, with SWV voltammograms recorded to assess the level of binding between Apt1_RC and UspA2. The analytical response of the biosensor was evaluated at each pH in multiple calibrations performed on different days. To quantify the sensitivity of the aptasensor in the detection of *E. coli* UspA2, calibration curves were plotted and the limit of detection (LOD) calculated. The LOD was computed as the concentration corresponding to X + 3σ, where X was the average value of the SWV blank signals (obtained by replacing *E. coli* UspA2 with PBS 1×) and σ the known standard deviation of consecutive SWV blank signal readings [33].

## 3. Results and Discussion

### 3.1. Characterization of UspA2

**Figure 1:**
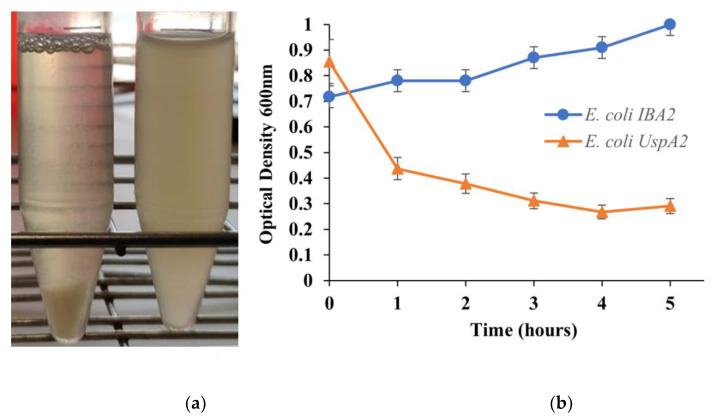
Illustration of a sedimentation assay. (**a**) Macroscopic analysis of auto-aggregation. Within a few minutes, *Escherichia coli* UspA2 (left tube) aggregates and settles at the bottom of the tube under static incubation, whereas the control culture of *E. coli* carrying the empty vector pASK IBA2 (*E. coli* IBA) (right tube) remains turbid. (**b**) Measurement of auto-aggregation. Culture samples were incubated statically and the OD_600nm_ value at the top of the culture tube was measured after 5 min of incubation. The reduction in turbidity at the top of the culture is a function of the OD_600nm_ value. Auto-aggregating bacteria settle at the bottom of the tube, resulting in a loss of turbidity (orange curve), whereas in negative control culture samples the reduction in turbidity is negligible (blue curve).

The use of pathogenic *M. catarrhalis,* which naturally expresses UspA2, was avoided throughout this study due to safety and practical concerns. Instead, a heterologous *E. coli* UspA2 was developed as a substitute for experiments involving UspA2. The recombinantly expressed UspA2 was evaluated by SDS-PAGE and characterized regarding its unique properties, such as auto-aggregation [23]. Auto-aggregation can be macroscopically observed when bacteria of the same type (pure culture) clump together and then settle at the bottom of culture tubes [34]. In the case of TAAs, when observed under an electron microscope, their head domains interact with each other in a zipper-like manner, making them stick together [19]. In a static culture of *E. coli* UspA2, after at least one hour of UspA2 induction, small, suspended clumps of auto-aggregated cells, looking similar to snow in a snow globe, were observed (Figure 1a). These clumps increased in size before finally settling to the bottom of the tube within a few minutes. The auto-aggregation of *E. coli* UspA2 was measured quantitatively by a sedimentation assay [23]. The reduction in OD_600nm_ of the *E. coli* UspA2 static cultures collected at various time points were plotted as a function of time (Figure 1b). An hour after UspA2 started being expressed, a reduction in OD_600nm_ of almost 50%, from ca. 0.85 to 0.44, was observed due to auto-aggregation and sedimentation of the cells and it continued to slightly reduce for the measured period. By contrast, the turbidity of the negative control (*E. coli* IBA) static cultures did not decrease as the cells did not aggregate in the absence of UspA2 and remained suspended; instead, it slightly increased over time due to the culture growth.

Regarding the SDS-PAGE, as can be verified in Figure S6, UspA2 cannot be observed in the gels. It is possible that the protein is not being expressed in sufficient amounts to be observed. The UspA2 monomer of the Bc5 strain has a size of 65.5 kDa and this protein is known to show aberrant migration in SDS-PAGE even when purified forms of the protein have been used and the cause of this has not been fully determined [35,36]. This effect is based on the high stability of TAA trimers, which leads to incomplete denaturation in the SDS sample buffer even after heating. The effect is also observed for other TAAs, e.g., *Yersinia* YadA [37] and *Bartonella* BadA [38]. Their coiled-coil structure may also affect their migration in the gel during electrophoresis [36]. In a study by Aebi et al., UspA2 derived from whole cell lysates of wild type bacteria, was observed in SDS-PAGE in a very high molecular weight oligomeric form of around 250 kDa [35].

### 3.2. Selection of Aptamers with High Affinity for UspA2 by Cell-SELEX

*E. coli* UspA2 was used as the target during each cycle of cell-SELEX to isolate aptamers with high-affinity to this adhesin. During the first cycle of cell-SELEX, an ssDNA library was incubated with live *E. coli* UspA2 cells in a positive selection, to reach the largest possible selection of ssDNA aptamers that bind the target cells. In the following cycles, bound sequences recovered from the preceding cycle were incubated with the target cells. During counter-selections or negative selection cycles (5th and 7th), *E. coli* UspA2 cells were replaced by *E. coli* IBA cells and the supernatant containing unbound sequences was recovered instead to eliminate any sequences recognizing surface molecules common to both *E. coli* strains. After each cycle, the recovered ssDNA was eluted and amplified by PCR (Figure S3) for use in the next selection cycle. A total of 8 cycles was considered optimal for isolating an aptamer pool with the highest affinity to target cells, as reported in recent studies [39,40].

High affinity aptamer ligands specific for UspA2 or *M. catarrhalis* have not been disclosed. Only a small number of antibody ligands with specificity to the family of UspA2 adhesins have been reported, primarily in search of vaccine candidates [24,25,41]. For example, a large semi-synthetic phage display library was used to select a panel of ten human antibodies that were able to identify UspA2 adhesins and distinguish between strains [41]. A few studies also identified UspA2-specific antibodies by traditional methods [24,25]. However, the antibodies identified in these studies were not characterized further with regards to their binding affinity to the UspA2 antigens. Neither were any translational studies performed for possible diagnostic or other applications. Furthermore, these studies are old and, remarkably, no recent efforts have been made to develop biorecognition elements towards UspA2 or *M. catarrhalis*.

### 3.3. Identification of Aptamer Candidates Aided by Bioinformatics

The aptamer pool obtained from the 8th cell-SELEX cycle was sequenced by NGS. NGS is highly advantageous as millions of reads can be performed, while only a few reads are possible with conventional sequencing. Thus, NGS, together with bioinformatics, makes it possible to analyze the immense number of sequences generated during cell-SELEX and select highly specific aptamers from among them [42,43]. From the data gathered by this state-of-the-art method, useful information such as the total number of reads, repetitions of each unique sequence and the rate of molecular enrichment can be derived [44].

**Figure 2:**
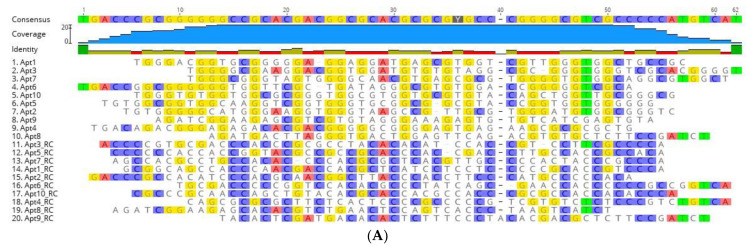

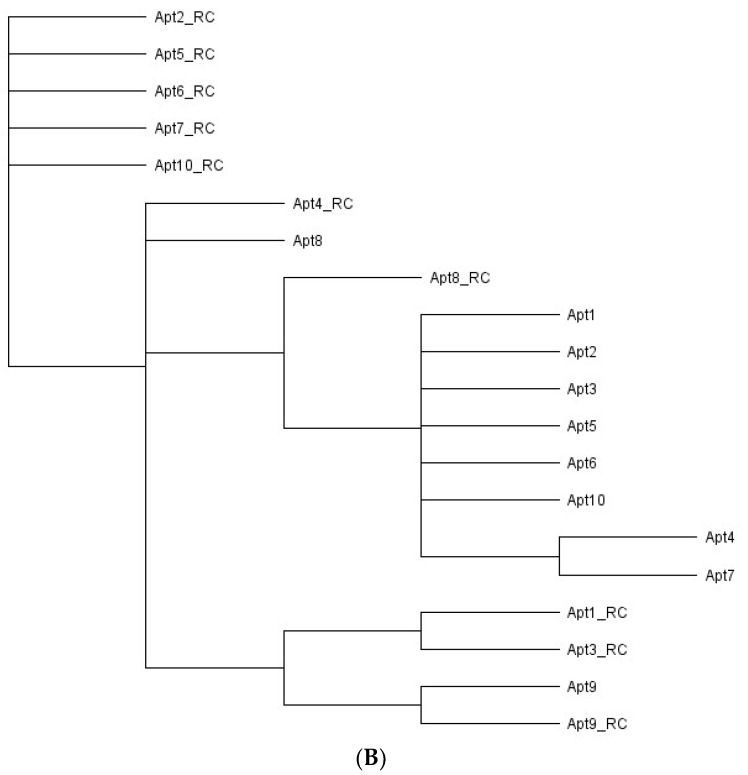
Sequence alignments and phylogenetic tree of the 20 aptamer sequences obtained after bioinformatic analysis. (**A**) Sequence alignments of the random region from the ten most frequent aptamer sequences and their reverse complement (RC) sequences obtained after NGS of the final aptamer pool of cell-SELEX. The regions of homology in the sequences are highlighted. (**B**) Phylogenetic relationship of the aptamer sequences using ‘Tree Builder’ in Geneious software. The relationship between the sequences was determined using a neighbor-joining model with no outgroups.

NGS results were further analyzed using the bioinformatics tool Geneious. Of the 7,788,762 sequences retrieved by NGS, 1,210,938 sequences remained after an initial filtration, the removal of the constant primer binding and adapter segments and removal of sequences outside the desired length (46–52 nt). Geneious was used to refine the sequences to obtain the 10 most repeated oligonucleotide sequences and their RC sequences, presented in Table 2 (displaying only the randomized region). The RC sequences were also considered, since PCR was used to amplify the ssDNA pool after each cell-SELEX cycle, causing the reverse complementary sequences to be introduced to the evolving aptamer pool in the following cycles and thus introducing the possibility that some of these RC aptamers could exhibit high-affinity to UspA2 and be retained in the final pool. Therefore, 20 sequences, namely the top 10 sequences and their RC, were further aligned to evaluate their homology within their variable core region (Figure 2A). Alignment of the N50 aptamer regions revealed limited homology and conservation. This suggests that most of these aptamers had unique binding sites on the target UspA2. A distribution of a high number of Gs in the forward sequences and Cs in the reverse complement sequences of the random regions is evident.

Analysis with regards to the phylogenetic relationship revealed that the selected aptamer sequences showed a distinct evolutionary tree (Figure 2B), where seven main families of related aptamers were identified (Apt1_RC and Apt3_RC; Apt1, 2, 3, 5, 6, and 10; Apt2_RC, Apt5_RC, Apt6_RC, Apt7_RC and Apt10_RC; Apt4_RC and Apt8; Apt9 and Apt9_RC; Apt8_RC; Apt4 and Apt7), showing that they do not have a very similar sequence for conserved nucleotides between them.

**Figure 3:**
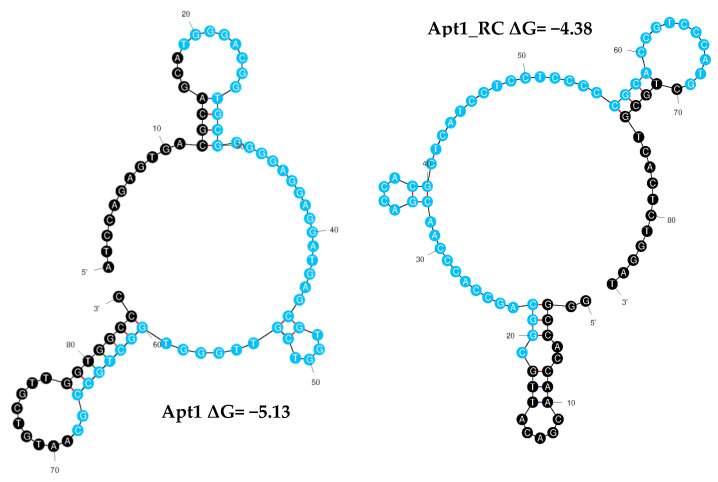
Secondary structures of finalized candidate aptamer sequences obtained from cell-SELEX. The Mfold web server was used to predict the secondary structures of Apt1 and Apt1_RC selected for in vitro characterization. To calculate the Gibbs free energy of the aptamer sequences, the constant primer regions were considered at 37 °C, 187 mM Na^+^ and 1.4 mM Mg^2+^. Random and primer regions are presented in blue and black, respectively.

Aptamers fold into unique conformations, which often include stems and loops. These structures are crucial for targeting molecule recognition [45]. Thus, the secondary structures of all the aptamer sequences (including the primer and random regions) were predicted using Mfold to examine their folding characteristics (Figure 3 and Table S3). Apt1_RC exhibited five loops in its structure, as well as Apt4_RC and Apt10_RC; Apt8_RC had seven loops, Apt10 six, Apt2_RC and Apt3_RC three each, and Apt9 had two. All the remaining aptamers Apt1, 2, 3, 4, 5, 6, 7 and 8 and Apt5_RC, Apt6_RC, Apt7_RC and Apt9_RC, exhibited four loop structures each.

From among the 20 aptamer sequences, Apt1 and Apt1_RC were chosen as the best candidate aptamers overall based on superior pool repeatability and conformation (Apt1_RC has the third highest number of loops out of all 20 sequences). Therefore, the sequences of Apt1 and Apt1_RC were synthesized with a FAM label to be used in the following experiments to characterize and analyze them further. No further tests were performed with the other aptamers.

### 3.4. Dissociation Constants of Candidate Aptamers with UspA2

**Figure 4:**
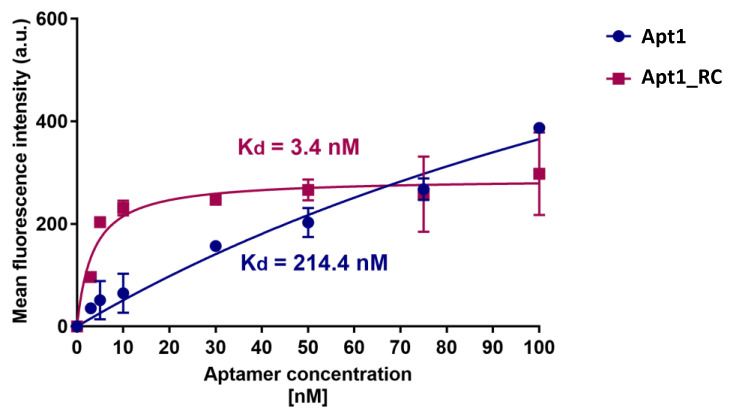
Determination of the equilibrium dissociation constant $\left(K_{d}\right)$ of candidate aptamers Apt1 and Apt1_RC. The titration curves of each aptamer with *E. coli* UspA2 cells are shown. The aptamers were labeled with fluorescein (FAM) and fluorescence was assessed with a fluorescence spectrophotometer. $K_{d}$ (nM) values were calculated using GraphPad Prism 7, set to a non-linear fit model with one-site non-competitive binding to the fluorescent population ratio at the given aptamer concentrations. a.u = arbitrary units.

The apparent *K_d_* values obtained by titration for the candidate aptamers Apt1 and Apt1_RC are shown in Figure 4. Apt1_RC demonstrated a low *K_d_* of 3.4 nM, which corresponds to a high level of affinity for *E. coli* UspA2, while Apt1 displayed a lower affinity towards *E. coli* UspA2, as suggested by a much higher *K_d_* of 214.4 nM. A titration of Apt1 and Apt1_RC with *E. coli* IBA was also performed as a negative control (Figure S7). The Apt1_RC presented a *K_d_* value of 22.7 nM during its interaction with the negative control *E. coli* IBA, a value significantly higher than the one reported during the incubation with *E. coli* UspA2 (6.9 times higher). Therefore, it is possible to conclude that the high affinity verified for Apt1_RC is related to UspA2. However, it is important to mention that the model that was used to calculate the *K_d_* values possibly does not consider the tendency of *E. coli* UspA2 to auto-aggregate.

Recent studies on the development of PoC platforms towards the detection of bacterial pathogens using aptamers have been reported, including *Shigella flexneri* (*K_d_* = 144 to 329 nM) [46], *Staphylococcus aureus* (*K_d_* = 16.5 nM and 14.47 nM) [47], *E. coli* (*K_d_* = 15.9 nM) [48], *Helicobacter pylori* (*K_d_* = 19.3 nM) [49], *Yersinia enterocolitica* (*K_d_* = 11 nM) [16] and *Salmonella typhimurium* for which a multi-aptamer probe was reported (*K_d_* = 3.09 nM) [50]. The binding affinity of Apt1_RC compares favorably with the binding affinity of other contemporary aptamer candidates selected for other bacterial pathogens. Thus, it can be concluded that Apt1_RC displays excellent affinity towards UspA2. Therefore, it was selected as the biorecognition element to be further used for the electrochemical detection of UspA2.

### 3.5. Assembly of the Aptasensor

**Figure 5:**
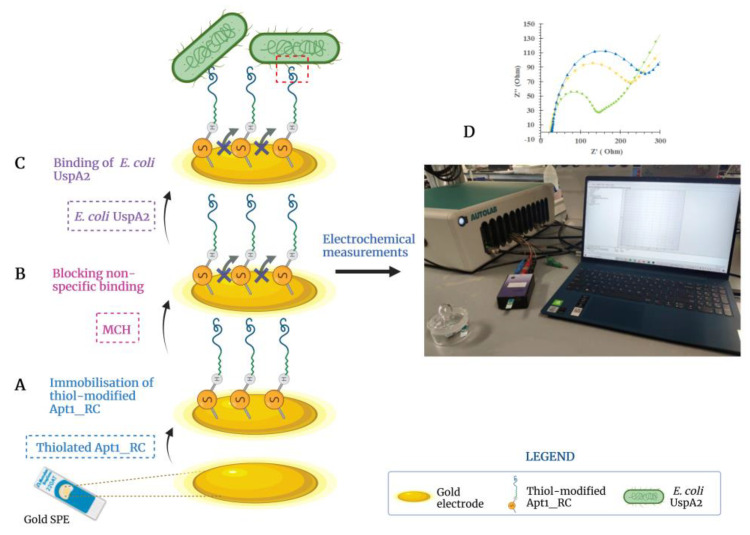
Illustration of the developed electrochemical biosensor to detect *Escherichia coli* UspA2. (**A**) Apt1_RC was immobilized on the gold electrode surface; (**B**) non-specific binding was blocked with 6-mercapto-1-hexanol (MCH); and (**C**) selective binding between *E. coli* UspA2 and Apt1_RC. (**D**) The electrochemical response is measured after each assembly step and during the analytical analysis of the performance of the aptasensor. Created with BioRender.com.

The steps involved in the assembly of the UspA2 aptasensor are shown in Figure 5. These include (i) cleaning of the Au-SPE; (ii) oligonucleotide immobilization (Figure 5A) and (iii) blocking of non-specific binding sites (Figure 5B). Additionally shown is the subsequent analytical stage during which the binding of *E. coli* UspA2 to the aptasensor occurs (Figure 5C), followed by electrochemical detection of the binding event (Figure 5D).

Proper immobilization of the biorecognition element is crucial for its activity and function and effective surface chemistry methods are required for this purpose. Typically, biorecognition elements have been modified with various chemical groups, such as silanes, thiols, amines and conducting polymers that covalently bind to functional groups on the working surface of biosensors [12]. More recently, nano-materials have also been effectively used on the electrode surface to increase the density of immobilized biomolecules, amplify sensor signals and electrical conductivity [51,52,53,54]. However, these modifications also increase the complexity and cost and are not essential for improving the sensitivity of a biosensor.

### 3.6. Electrochemical Characterization of the Assembled Aptasensor

**Figure 6:**
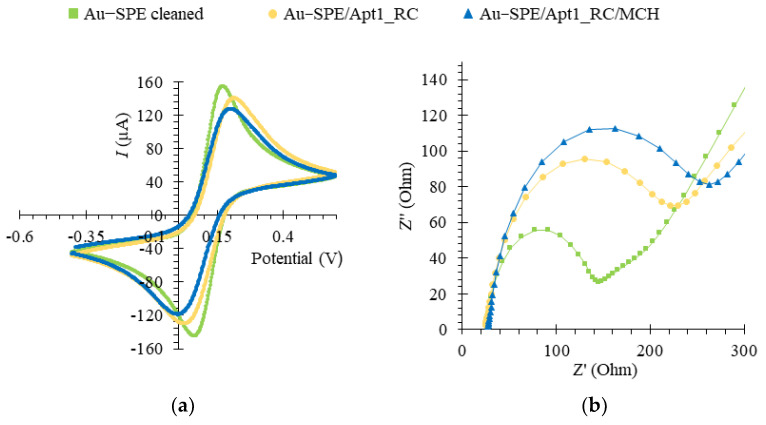
Electrochemical assays to assess the assembly of the aptasensor upon functionalization of the Au-SPE surface, in 5.0 × 10^−3^ M [Fe (CN)_6_ ]^3−^ and 5.0 × 10^−3^ M [Fe (CN)_6_]^4−^ solution, prepared in phosphate buffer, pH 7.4. Cyclic voltammetry (CV) (**a**) and electrochemical impedance spectroscopy (EIS) represented by a Nyquist plot (**b**).

EIS and CV assays were used to follow-up on the chemical modification of the Au-SPE surface during the aptasensor assembly. The redox probe [Fe (CN)_6_]^3−^/[Fe (CN)_6_]^4−^ at pH 7.4 was selected to monitor the electron transfer properties, due to possessing a fast electron transfer rate [32,55]. A voltammogram corresponding to the CV analysis of the aptasensor assembly is shown in Figure 6a. The decrease in the quasi-reversible cathodic/anodic peak currents of the Au-SPE upon immobilization of Apt1_RC on its surface (Au-SPE/Apt1_RC) compared to the bare, cleaned Au-SPE surface is due to an increased charge-transfer resistance caused by electrostatic repulsion between a negatively charged redox couple and the negatively charged phosphate backbone of Apt1_RC [56]. Similarly, a further decrease in the peak currents is observed upon modification with MCH (Au-SPE/Apt1_RC/MCH) due to MCH blocking of the free Au surface. These results indicate that chemical changes occurred at the Au-SPE surface, i.e., the successful immobilization of Apt1_RC.

In the related EIS assays, the charge-transfer resistance (*R*_ct_) is inversely proportional to the rate of electron transfer and was determined by fitting data to a Randles equivalent circuit, which characterizes the physicochemical process occurring at the Au surface. In the circuit, the double layer capacitance (*C*dl) is replaced with a constant phase element (non-ideal capacitance), in parallel with *R*_ct_ and a Warburg element that models diffusion [57]. The EIS is expressed as a Nyquist plot, as shown in Figure 6b. The diameter of the semicircle corresponded to the *R*_ct_ and the linear portion to the process of diffusion of ions. For the bare Au electrode, it was possible to observe a small semi-circle domain with a straight line, suggesting the electron transfer process was limited by mass diffusion of ions. The *R*_ct_ increased considerably upon immobilization of the Apt1_RC probe on the clean Au surface. It increased further with subsequent blocking of the free Au surface with MCH, thus, confirming the CV analysis. Figure S8 shows the Bode phase plot corresponding to the EIS data of the aptasensor assembly, which also confirms the correct assembly of the aptasensor. Additionally, the biosensor setup proved to be highly reproducible with a relative standard deviation (RSD) of 0.63%, *n* = 3 for CV and 7.07% for EIS measurements, and displayed excellent repeatability with an RSD of 0.60% for CV and 1% for EIS. The detection times for one sample were 2 and 4 min for the CV and EIS methods, respectively.

### 3.7. Detection of E. coli UspA2 by the Aptasensor

**Figure 7:**
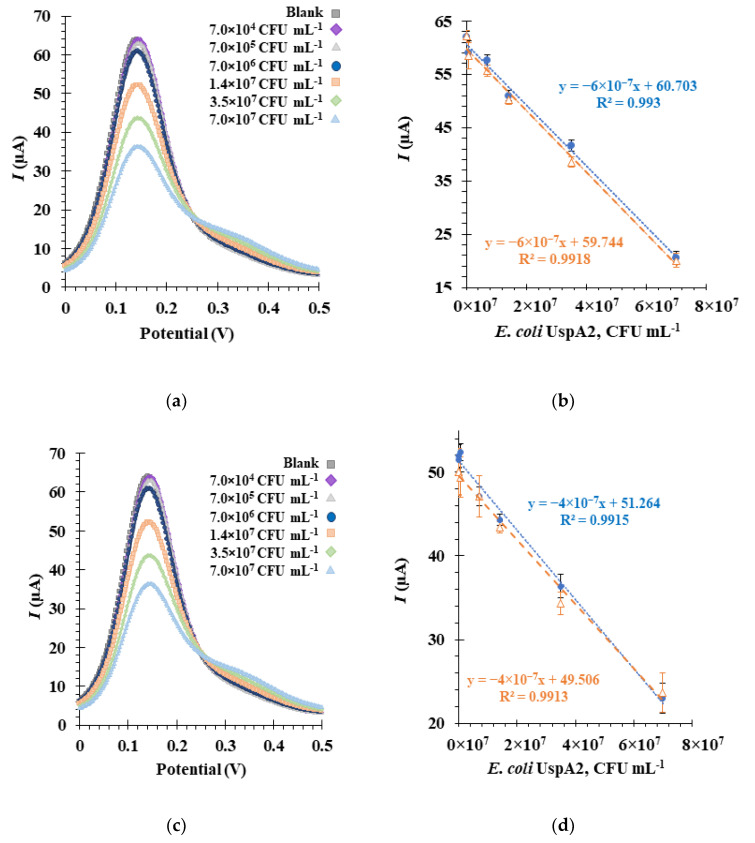
Electrochemical assays to assess the analytical response of the aptasensor in square wave voltammetry (SWV) (**a**,**c**), and the corresponding calibration curves (**b**,**d**), in 5.0 × 10^−3^ M [Fe (CN)_6_]^3−^ and 5.0 × 10^−3^ M [Fe (CN)_6_]^4−^ in phosphate buffer, pH 7.4. The aptasensors functionalized with Apt1_RC were incubated sequentially with increasing concentrations of inactivated *Escherichia coli* UspA2 cells in a buffer at pH 5.0 (**a**,**b**) or at pH 7.4 (**c**,**d**). The orange and blue plot lines (**b**,**d**) represent calibration curves performed on different days.

SWV was used to monitor the electrochemical detection of *E. coli* UspA2 by the Apt1_RC-modified biosensor (Figure 7). SWV has certain advantages over other voltammetric techniques, such as CV, differential pulse voltammetry (DPV) and chronoamperometry (CA), including higher sensitivity, faster analysis time and the ability for significant reduction of capacitance currents [58]. In the SWV technique, current intensity (I) values obtained with the redox probe were recorded in response to changes in concentrations of *E. coli* UspA2. Inactivated *E. coli* UspA2 cultures maintained at two different pH values (pH 5.0 in MES buffer and pH 7.4 in PBS 1× buffer) were tested to determine the effect of pH on the biosensor detection sensitivity to UspA2 [59]. In general, the SWV voltammograms obtained from the calibration curves revealed that peak currents decreased with increasing concentrations of *E. coli* UspA2 at both pH values (Figure 7a,c). This can be attributed to the increase in charge transfer resistance at the Au-SPE surface. The selected SWV data of the biosensor response at each pH during calibration assays is shown in Figure 7b,d. The orange and blue plot lines represent calibration curves generated on different days. The electrochemical response proved to be highly reproducible (RSD 2.7%, *n* = 3 in SWV), and repeatability was excellent (RSD 0.13%, *n* = 3 in SWV). The detection time for one sample was 1 min for the SWV method.

From the calibration curve at pH 5.0 (Figure 7b), a linear correlation could be established for a concentration range from 7.0 × 10^4^ CFU mL^−1^ to 7.0 × 10^7^ CFU mL^−1^, with an average slope of −6.0× 10^−7^ μA mL CFU^−1^ and a square correlation coefficient superior to 0.99. The relative standard deviation of these assays was ~2.5%. The LOD was calculated and found to be 4.0 × 10^4^ CFU mL^−1^.

As observed from the voltammogram at pH 7.4 (Figure 7c), the lowest concentration tested yielded an I signal similar to the blank. A change/decrease in the I signal was only observed at a concentration of approximately 7.0 × 10^5^ CFU mL^−1^, which is tenfold higher than in pH 5.0 experiments. Thus, from the calibration curve, a linear trend could be established from 7.0 × 10^5^ to 7.0 × 10^7^ CFU mL^−1^ (Figure 7d) with an average slope of −4.0 × 10^−7^ μA mL CFU^−1^ and a square correlation coefficient superior to 0.99. The LOD and the relative standard deviation of these assays were 1.6 × 10^5^ CFU mL^−1^ and ~2.7%, respectively. Therefore, it can be concluded that the aptasensor functions are more sensitive at pH 5.0 based on the LOD and slope values [16]. This analysis suggests, importantly, that factors in the sensory environment could play an important role in determining the detection capabilities of the aptasensor.

Except for one recent work [60], no PoC platforms, such as biosensors or assays, have been developed for the detection of *M. catarrhalis*. In the latter, Lee et al. [60] developed a colorimetric and fluorogenic probe that could detect *M. catarrhalis* with a LOD of 10^3^ CFU mL^−1^ by visual inspection within 10 min. The authors used a novel meso-ester-substituted boron dipyrromethene dye that sensitively detects the C4-esterase activity of *M. catarrhalis.* Although this method is advantageous in terms of its simplicity, novelty and sensitivity, it lacks selectivity. Other pathogenic bacteria, such as *Staphylococcus aureus, Listeria monocytogenes* and *E. coli,* which also exhibit esterase activity, were tested and produced a colorimetric response. Therefore, additional laboratory testing would be required to confirm the specific presence of *M. catarrhalis* in the samples. In contrast, due to the high affinity for UspA2, the aptasensor developed here is believed to be specific for *M. catarrhalis,* although further experiments with non-target bacterial species are required to prove this. Additionally, the straightforward design of the aptasensor eliminates the need for redundant components and reagents, resulting in reduced complexity, lower costs, ease of use and rapid detection.

Since *M. catarrhalis* infections mostly affect children, this aptasensor could be used for on-site diagnosis in cases of pediatric ear infections. The aptasensor could also potentially help distinguish between *M. catarrhalis* and *Neisseriaceae* infections, which are often mistaken for each other in culture.

## 4. Conclusions and Future Perspectives

A novel, label-free electrochemical aptasensor of simple design was developed to detect the UspA2 adhesin protein of *M. catarrhalis*. PoC-based studies to detect *M. catarrhalis* are virtually non-existent, despite its status as a pathogen of concern. Therefore, the developed aptasensor could serve as a much-needed solution towards implementing the POC diagnosis of *M. catarrhalis*. A major advantage of this work is the simplicity of the aptasensor design while still providing good detectability. With the evaluation of an aptamer bioreceptor selected through cell-SELEX, it was demonstrated that ligands other than antibodies can be used for sensitive binding to a target, which diversifies the viable options of the ligands /bioreceptors. The highly sensitive and reproducible detection of *E. coli* UspA2 with an LOD of 4.0 × 10^4^ CFU mL^−1^ indicates that this aptasensor has the potential to detect clinically relevant amounts of bacterial *M. catarrhalis* cells.

To develop this work further in the future, the detection of real *M. catarrhalis* bacteria by the aptasensor can be tested, as well as with other strains of pathogenic and non-pathogenic bacteria. Additionally, the aptasensor can be evaluated with biological matrices (e.g., serum and plasma) and ultimately using real patient samples (e.g., fecal matter and body fluids) to be potentially clinically relevant. Finally, an innovative and straightforward diagnostic workflow is conceivable, comprising the aptasensor developed herein together with a previous sample enrichment step using collagen-modified magnetic nanoparticles [61] to improve the sensitivity of the aptasensor and serve as a model workflow to be replicated for the development of PoC diagnostic platforms for other bacterial pathogens.

## Figures and Tables

**Table 1:** Conditions used in the 8 cell-systematic evolution of ligands by exponential enrichment (cell-SELEX) selection cycles performed to isolate aptamers with high affinity to UspA2 from *Moraxella catarrhalis*.

Cycles	*E. coli* UspA2 (µL)	Incubation (Minutes)	Washes (after Selection)	PCR (Cycles)
1	500	60	2	30
2	500	50	2	25
3	400	45	3	25
4	400	40	3	18
5	400 (counter-selection)	40	-	20
6	350	35	4	22
7	350 (counter-selection)	35	-	13
8	350	30	4	15

**Table 2:** Selected aptamer sequence candidates. Ten most repeated aptamer sequences and their reverse complement (RC) sequences obtained after next-generation sequencing (NGS) of the final (8th) cell-SELEX cycle. Only the random region part of sequences is shown.

Aptamer	Sequences (5′–3′)	Copies
Apt1	TGGGACGGTGCGGGGGAGGAGGATGAGCGTGGTCGTTGGGTGGCTGCCGC	16,130
Apt1_RC	GCGGCAGCCACCCAACGACCACGCTCATCCTCCTCCCCCGCACCGTCCCA	
Apt2	TGTGGGGGCATGGGAAGGTGGGTAAGCCGTTGCGTGGGATGTGGCGGGTC	13,652
Apt2_RC	GACCCGCCACATCCCACGCAACGGCTTACCCACCTTCCCATGCCCCCACA	
Apt3	TGGGGCGAAGGACGGTGGATGTGTGTAGGCGCGGGTGGGTCGCACGGGGT	12,992
Apt3_RC	ACCCCGTGCGACCCACCCGCGCCTACACACATCCACCGTCCTTCGCCCCA	
Apt4	TGACAGACGGGAGAGACACGACGGGGGCGGGGAGTGAGAAGCGCGCGCTG	12,694
Apt4_RC	CAGCGCGCGCTTCTCACTCCCCGCCCCCGTCGTGTCTCTCCCGTCTGTCA	
Apt5	TGTGGCGGTGGCAAGGTCGGTGGGTGCGGCGGCGTACCGGTGGTGGGGGG	9598
Apt5_RC	CCCCCCACCACCGGTACGCCGCCGCACCCACCGACCTTGCCACCGCCACA	
Apt6	TGACCGGCGGGGGGTGGTTCGCTGATAGGGCGTGTGGACCGGGGGTCGCA	9107
Apt6_RC	TGCGACCCCCGGTCCACACGCCCTATCAGCGAACCACCCCCCGCCGGTCA	
Apt7	TGGGCGGGTAGTGGGGCAACGTGAGCGCGTGGGGTGTGGCAGGCGTGGCT	8004
Apt7_RC	AGCCACGCCTGCCACACCCCACGCGCTCACGTTGCCCCACTACCCGCCCA	
Apt8	AGATGACTTAGGGTGACTGGAGTTCAGACGTGTGCTCTTCCGATCT	7558
Apt8_RC	AGATCGGAAGAGCACACGTCTGAACTCCAGTCACCCTAAGTCATCT	
Apt9	AGATCGGAAGAGCGTCGTGTAGGGAAAGAGTGTGTCATCGAGTGTA	7229
Apt9_RC	TACACTCGATGACACACTCTTTCCCTACACGACGCTCTTCCGATCT	
Apt10	TGGGTGTGGTGGCGCGGGTGGCGTGGTGCGTGTACAGCTGGTTGCGGGCG	7129
Apt10_RC	CGCCCGCAACCAGCTGTACACGCACCACGCCACCCGCGCCACCACACCCA	

## Data Availability

Not applicable.

## Supplementary Materials

The following supporting information can be downloaded at: https://www.mdpi.com/article/10.3390/bioengineering10020178/s1, Figure S1: Steps involved in molecular cloning by advanced quick assembly (AQUA); Figure S2: Schematic illustration of cell-SELEX cycles; Figure S3: PCR amplification gels after each of the eight cell-SELEX cycles; Figure S4: Au electrode; Figure S5: Representation of the Randles’ equivalent circuit; Figure S6: SDS–PAGE gel of *E. coli* UspA2 protein lysates; Figure S7: Determination of the equilibrium dissociation constant $\left(K_{d}\right)$ of the candidate aptamers; Figure S8: Bode phase plot of the biosensor assembly. Table S1: Primers for PCR amplification of the UspA2 gene and the pASK_IBA2 plasmid; Table S2: Oligonucleotide sequences used in the aptamer selection, validation process and immobilization on the biosensor; Table S3: Predicted secondary structures of the other nine highest-copy aptamers and their reverse complement sequences.
