# Supplementary material for: Aptasensor for the Detection of Moraxella catarrhalis Adhesin UspA2
Source: Bioengineering (Basel). 2023 Jan 31;10(2):178. doi: 10.3390/bioengineering10020178 (PMC9951875; doi:10.3390/bioengineering10020178)
Supplement: Supplementary file 1 [file bioengineering-10-00178-s001.zip › bioengineering-2144966-SI.pdf]

bioengineering

MDPI

Supplemmentary Material

Aptasensor for the detection of Moraxella Catarrhalis adhesin UspA2

Maria G. Sande, Débora Ferreira, Joana L. Rodrigues, Luís D. R. Melo, Athanasios Saragliadis, Dirk Linke, Felismina T. C. Moreira, M. Goreti F. Sales and Ligia R. Rodrigues

Corresponding author: Tel: (+351) 253601978, Email: lrmr@deb.uminho.pt

Table S1. Primers for PCR amplification of the UspA2 gene and the pASK_IBA2 plasmid (forward (FW) and reverse (REV) primers)

Primer name	Primer sequence[a]
FW _UspA2	AAATCTAGATAACGAGGGCAAAAA**ATG**AAAACCATGAAACTTCTCCCCC
REV _UspA2	CATTTTTCACTTCACAGGTCAAGC**TTA**GAACTCGTAATTCACACCGATGTTATAAG
FW _pASK_IBA2	GCTTGACCTGTGAAGTGAAAAATG
REV _pASK_IBA2	TTTTTGCCCTCGTTATCTAGATTTTTG

[a]Start and stop codons in **bold**; underlined sequences are homologous to pASK_IBA2 (IBA BioTAGnology).

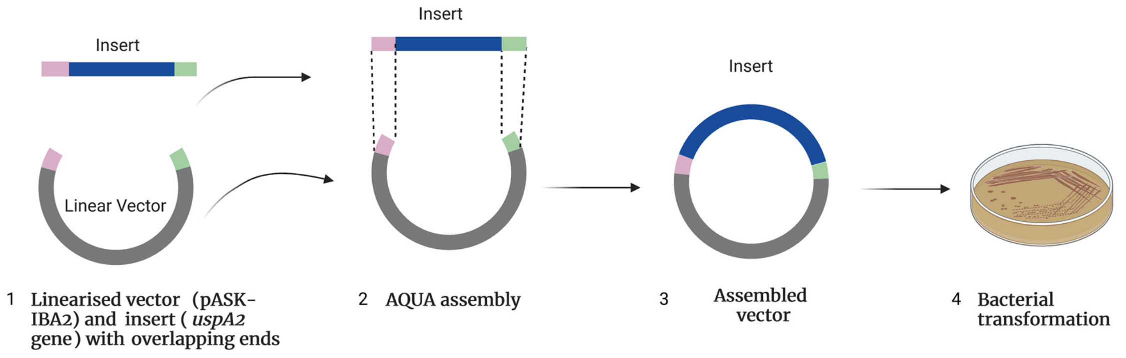

Figure S1. Steps involved in molecular cloning by advanced quick assembly (AQUA). This method was used to clone *uspa2* gene into the pASK-IBA2 vector. The vector: insert ratio used was 1:3. The assembled vector was transformed into *E. coli* Top10 competent cells. Created with BioRender.com.

Table S2. Oligonucleotide sequences used in the aptamer selection, validation process and immobilization on the biosensor.

**Name**	**Sequence (5'- 3')**
ssDNA Library	ATCCAGAGTGACGCAGCA -**N50**- AATGTCGTTGGTGGCCC
Forward Primer	ATCCAGAGTGACGCAGCA
Reverse Primer	AATGTCGTTGGTGGCCC
Aptamer 1	ATCCAGAGTGACGCAGCA**TGGGACGGTGCGGGGGAGGAGGATGAGCGT GGTCGTTGGGTGGCTGCCGC**AATGTCGTTGGTGGCCC
FAM-Aptamer 1	FAM-ATCCAGAGTGACGCAGCA**TGGGACGGTGCGGGGGAGGAGGATGAGCGT GGTCGTTGGGTGGCTGCCGC** AATGTCGTTGGTGGCCC
Aptamer 1_RC	GGGCCACCAACGACATT**GCGGCAGCCACCCAACGACCACGCTCATCCTCC TCCCCCGCACCGTCCCA**TGCTGCGTCACTCTGGAT
FAM-Aptamer 1_RC	FAM-GGGCCACCAACGACATT**GCGGCAGCCACCCAACGACCACGCTCATCCTCC TCCCCCGCACCGTCCCA**TGCTGCGTCACTCTGGAT
Thiol-Aptamer 1_RC	Thiol-C6-GGGCCACCAACGACATT**GCGGCAGCCACCCAACGACCACGCTCATCCTCC TCCCCCGCACCGTCCCA**TGCTGCGTCACTCTGGAT

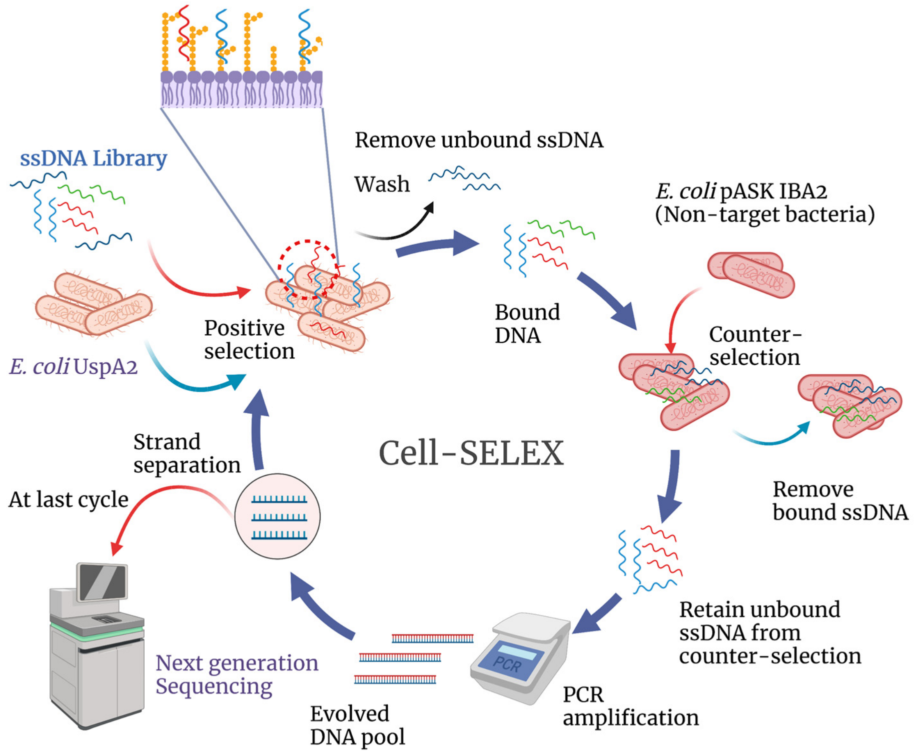

Figure S2. Schematic illustration of cell-SELEX cycles. Shown are the steps involved in the cell-SELEX cycles performed to isolate an aptamer pool with high-affinity towards UspA2 adhesin. Eight evolution cycles were performed, starting with a ssDNA random library in the first cycle. The aptamer pool from the 8th final cycle was sequenced by next generation sequencing (NGS). The image was reproduced with minor changes from Sande *et al*. [16]; Created with BioRender.com

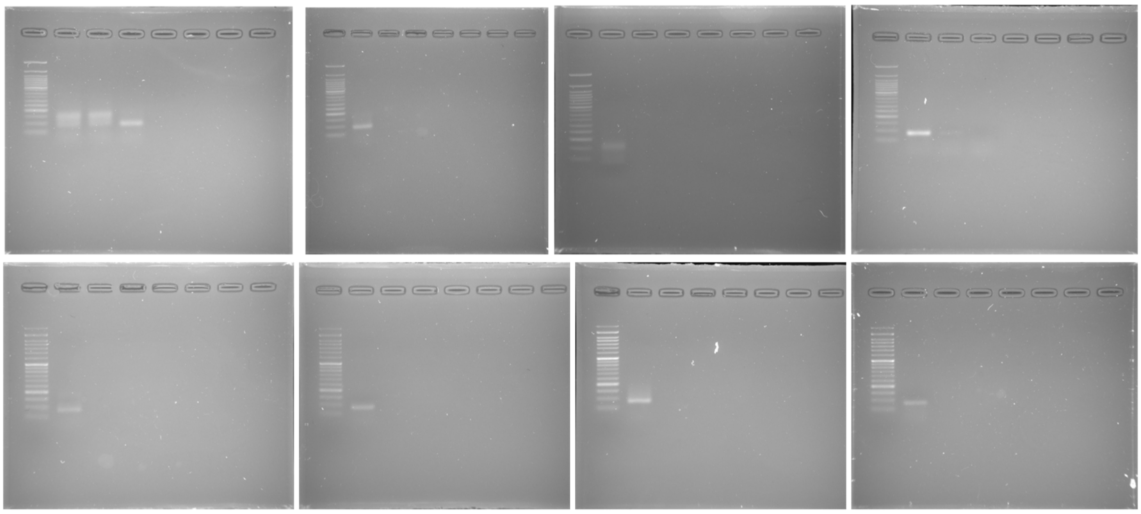

Figure S3. PCR amplification gels after each of the eight cell-SELEX cycles. Small amount of PCR product (85 nt) analyzed in an agarose gel (3%) after each PCR reaction to confirm the presence of the dsDNA at the correct size (Top: 1st -4th cycle gel, Bottom: 5th -8th cycle gel; In the 1st gel, the band corresponds to the fourth lane).

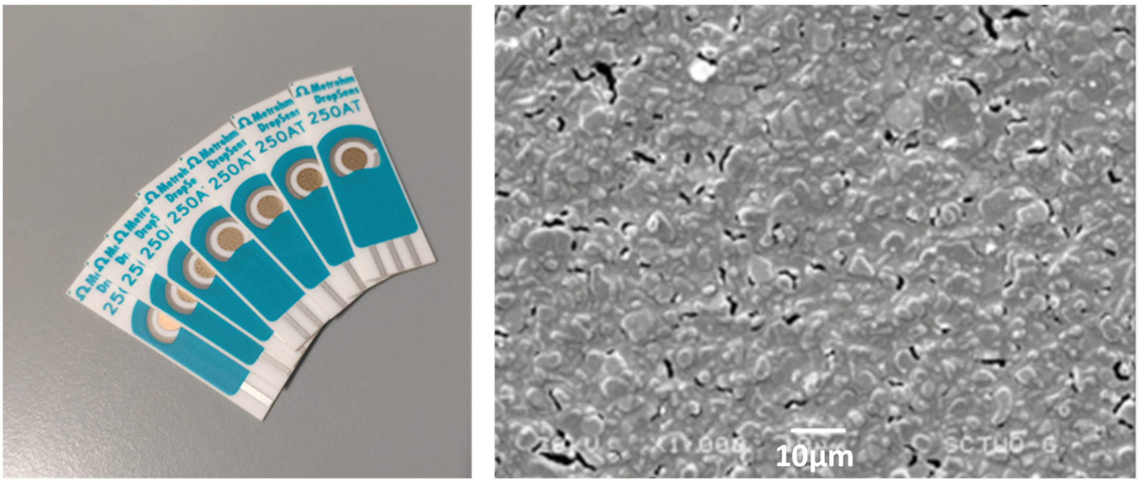

(A) **(B)**

Figure S4. Electrode. (A) Disposable screen-printed Gold electrodes (250 AT) purchased from DropSens were used in this study. (B) Scanning electron microscope image of the bare gold (Au) working electrode surface. (Image retrieved from Metrohm Dropsens https://www.dropsens.com/en/pdfs_productos/new_brochures/gold_electrodes.pdf)

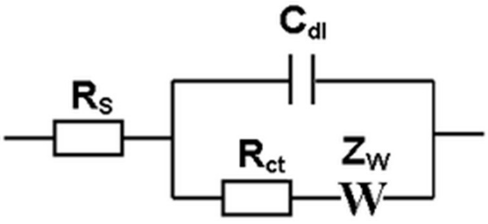

Rs - Double layer capacitance

Cdl - Ionic resistance

Rct - Charge transfer resistance

Zw - Warburg impedance

Figure S5 - Representation of the Randles' equivalent circuit.

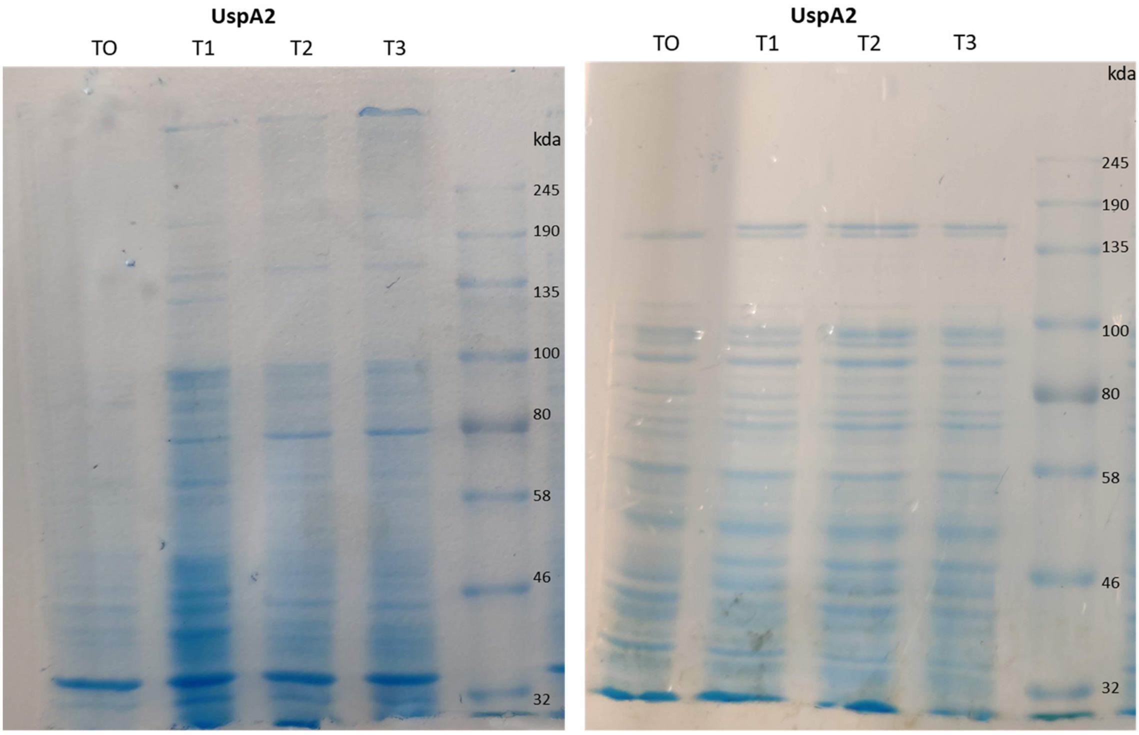

(A) **(B)**

Figure S6. SDS–PAGE gel of *E. coli* UspA2 protein lysates. (A) and (B) correspond to soluble and insoluble phases, respectively. The rightmost lane corresponds to the Color Prestained Protein Standard, broad range (11–245 kDa) (NEB). T0 corresponds to cell extract before protein expression was induced (10 µg). T1, T2 and T3 correspond to cell extracts –1, 2 and 3 hours after protein expression was induced (10 µg), respectively. UspA2 was expected at size 65.5 kDa or 250 kDa in monomer or oligomer form, respectively.

Table S3. Predicted secondary structures of the other nine highest-copy aptamers and their reverse complement sequences. The presented predicted secondary structures were the ones with lowest ΔG, i.e. the highest stability using the temperature 37 °C, 137 mM Na^+ and 1.4 mM Mg^{2+} (calculated using the Mfold webserver). Random regions are presented in blue. The aptamers sequence size range is 81-85 nt (≈ 6.33-7.55 kDa) considering both the primer and random regions.

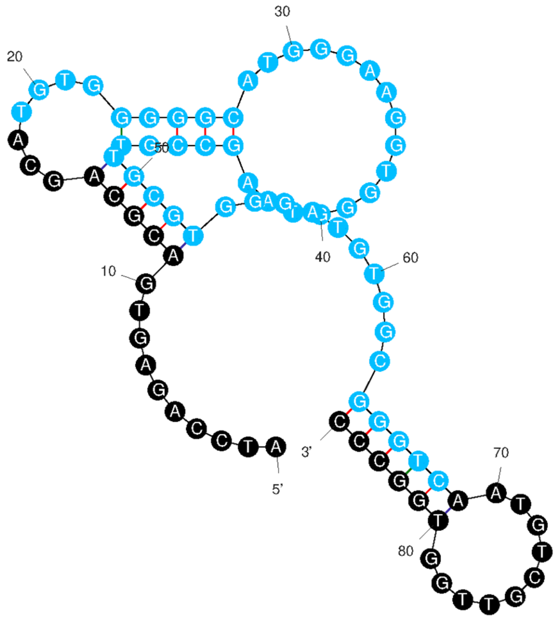 **Apt2, ΔG = -3.45**	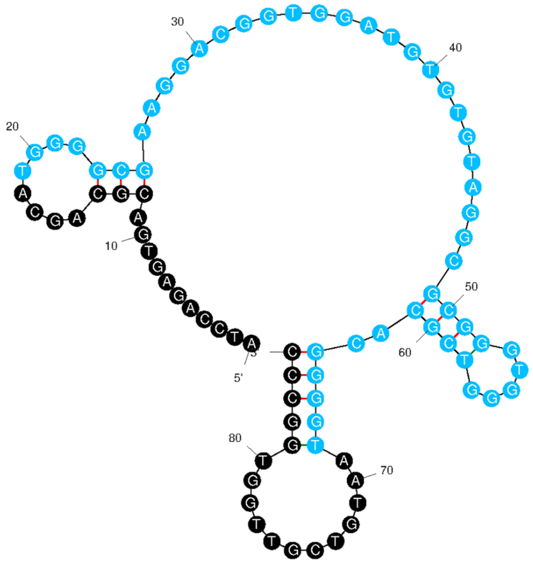 **Apt3, ΔG = -4.52**
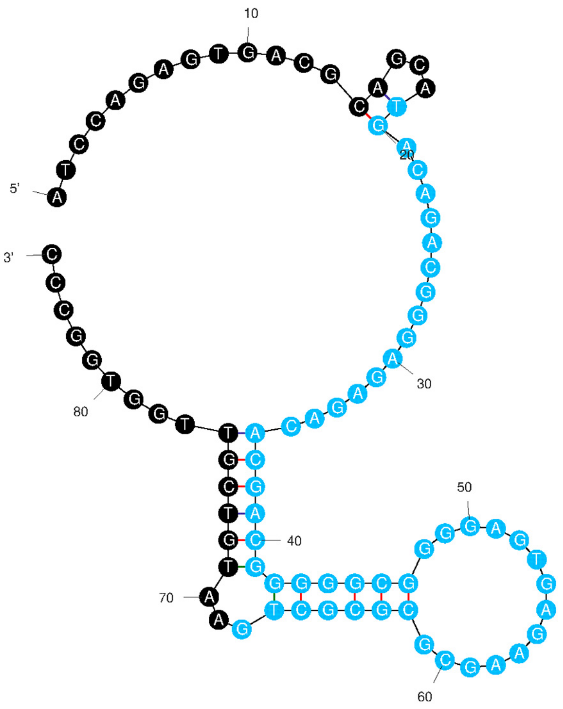 **Apt4, ΔG = -4.07**	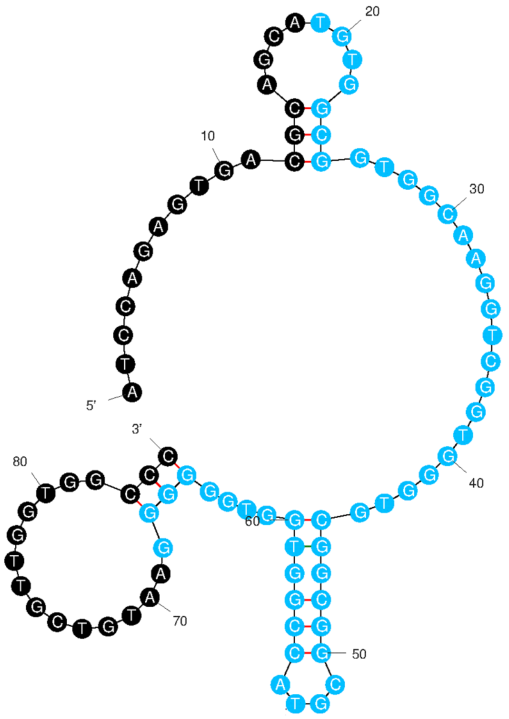 **Apt5, ΔG = -5.01**

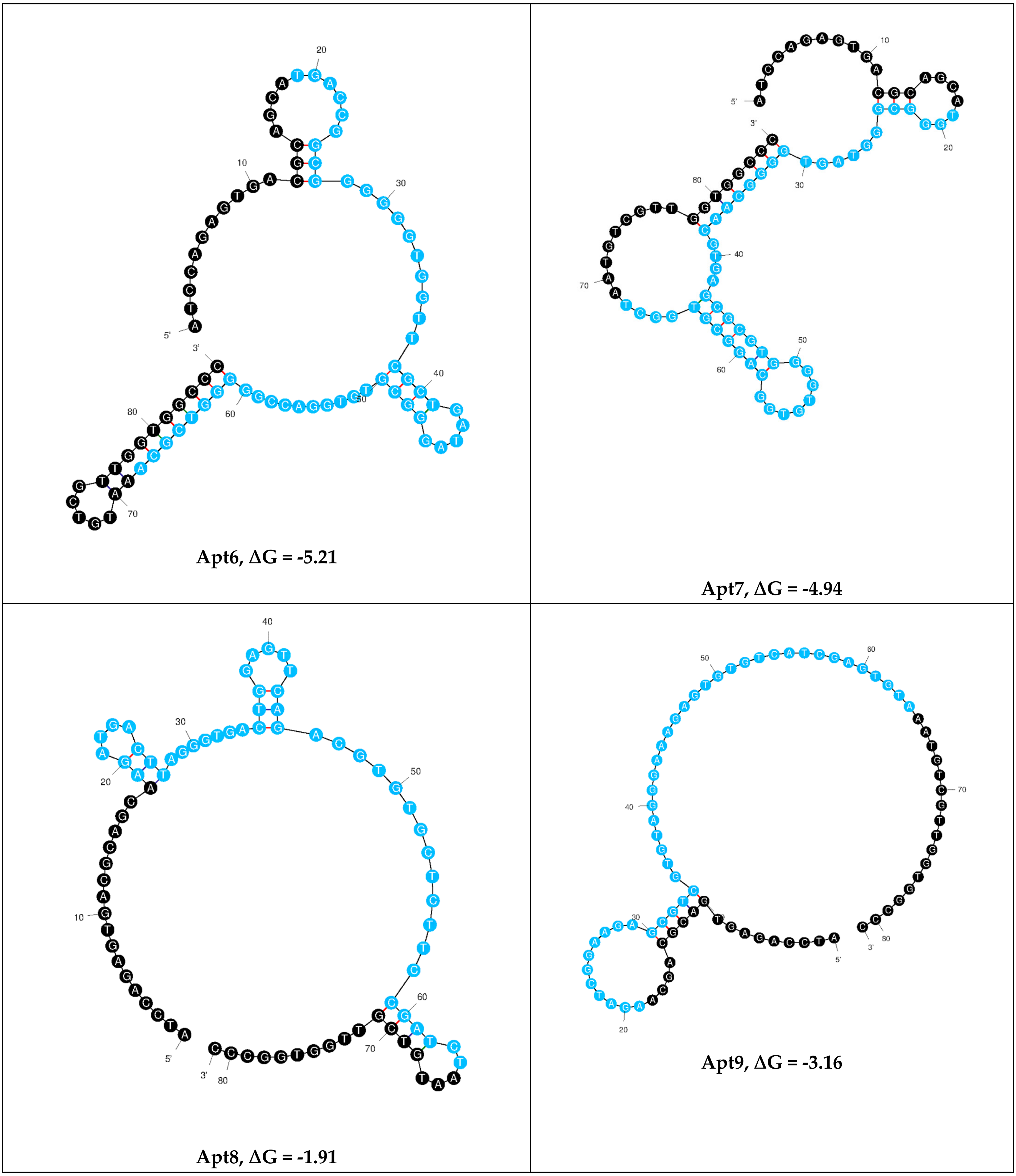
Apt6, ΔG = -5.21
Apt7, ΔG = -4.94
Apt8, ΔG = -1.91
Apt9, ΔG = -3.16

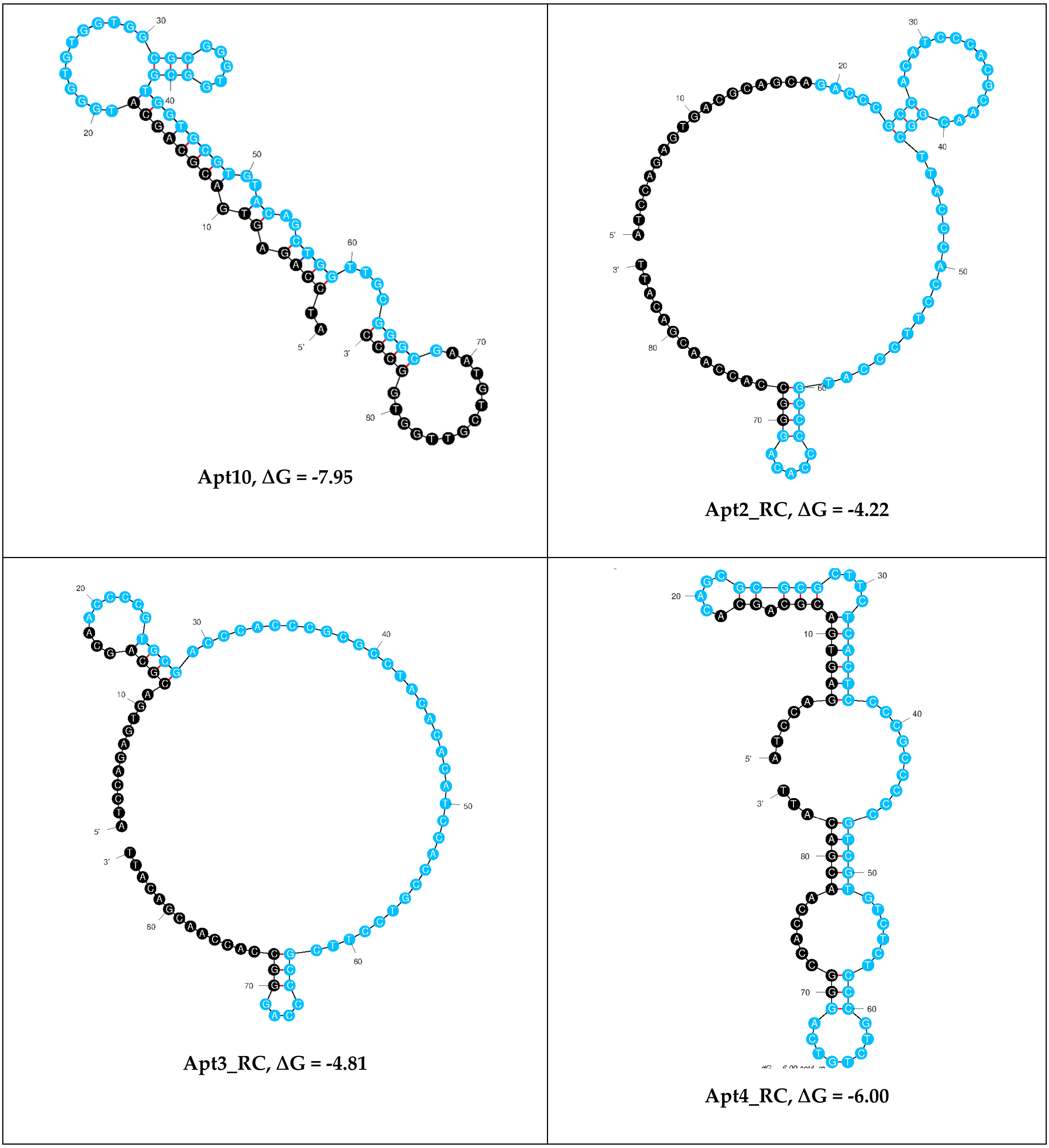

Apt10, ΔG = -7.95

Apt2_RC, ΔG = -4.22

Apt3_RC, ΔG = -4.81

Apt4_RC, ΔG = -6.00

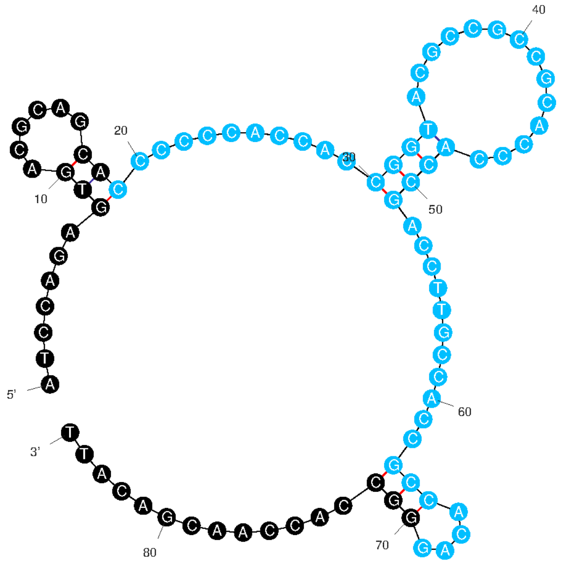

Apt5_RC, ΔG = -5.4

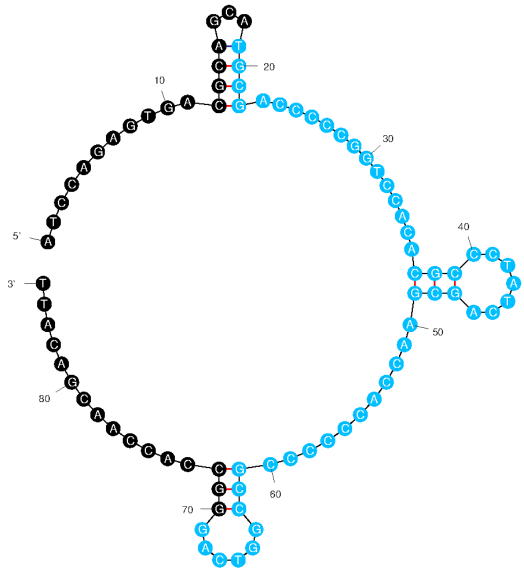

Apt6_RC, ΔG = -7.81

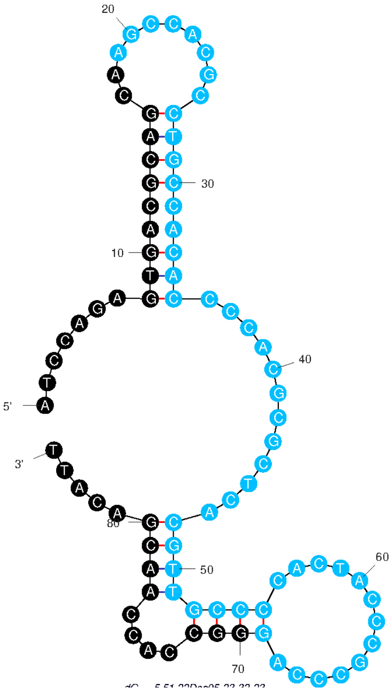

Apt7_RC, ΔG = -5.51

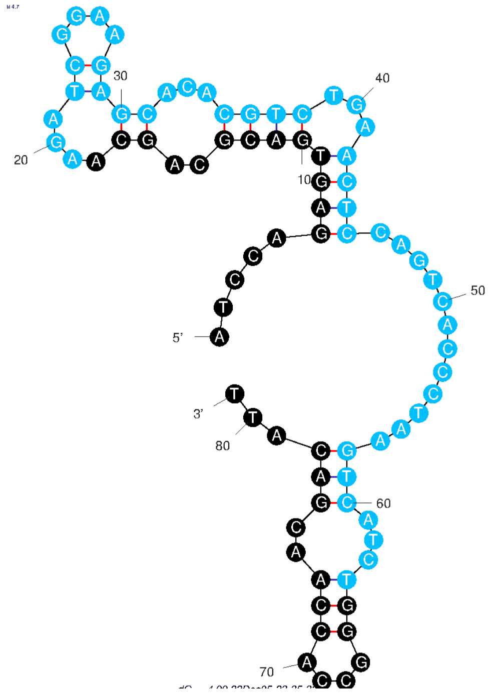

Apt8_RC, ΔG = -4.99

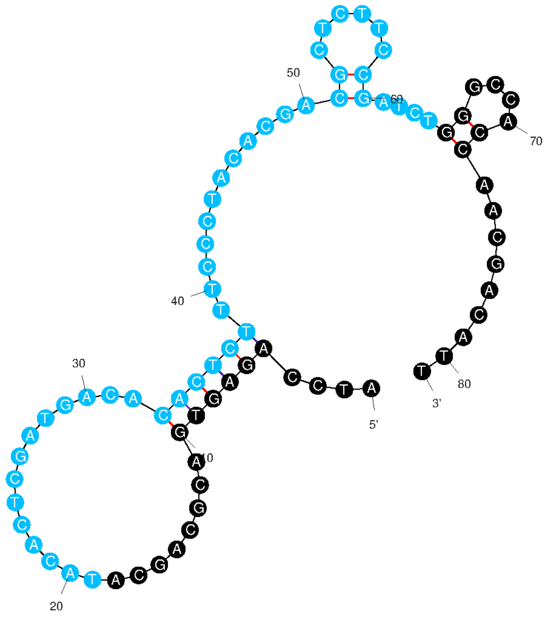

Apt9_RC, ΔG = -2.73

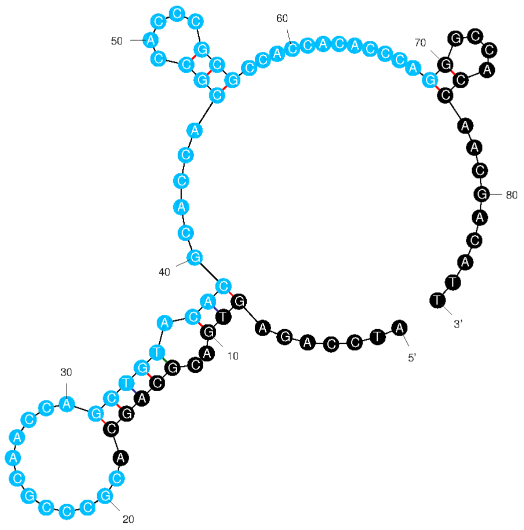

Apt10_RC, ΔG = -4.66

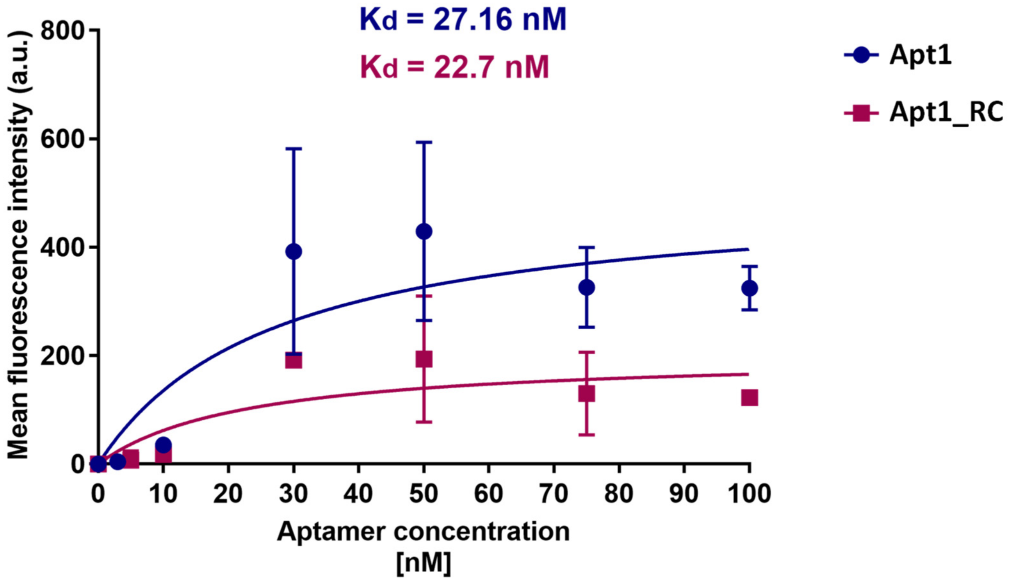

Figure S7. Determination of the equilibrium dissociation constant (K_d) of the candidate aptamers. The binding curves of aptamers Apt1 and Apt1_RC with *E. coli* IBA cells (negative control) are shown. Cell samples were incubated with increasing concentrations of fluorescein (FAM)-labelled aptamers and the fluorescence was calculated using a fluorescence spectrophotometer. K_d (nM) were calculated using GraphPad Prism 7, under the non-linear fit model, one-site non-competitive binding to fluorescent population ratio at used aptamer concentrations. a.u = arbitrary units.

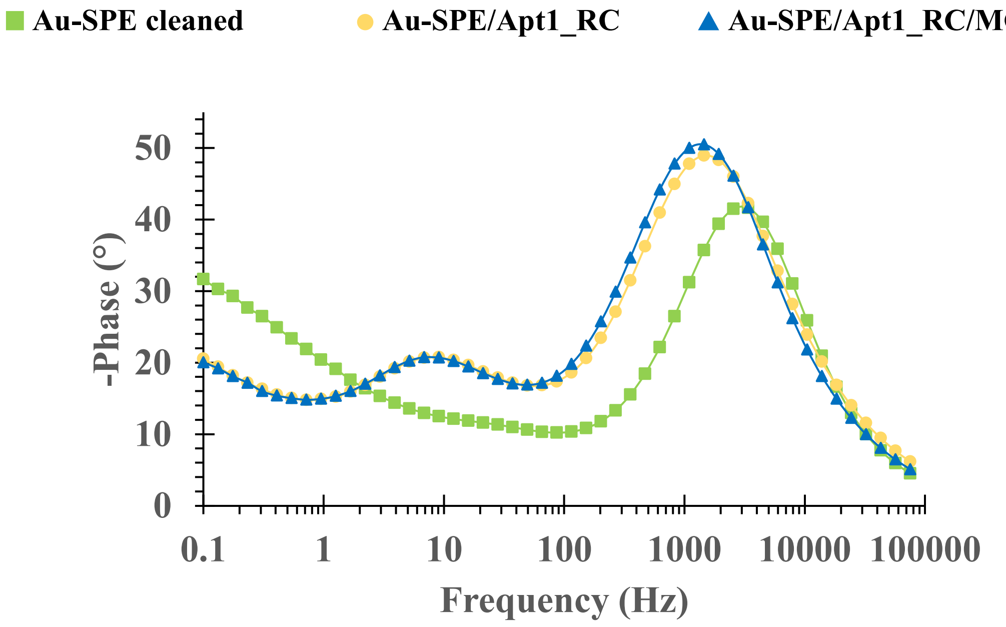

Figure S8. Bode phase plot of the biosensor assembly. Electrochemical assays for the construction of the biosensor by gold (Au) surface modification, in 5.0 x 10^{-3} M [Fe $(CN)_6$ $]^{3-}$ and 5.0 x 10^{-3} M [Fe $(CN)_6]^{4-}$ solution, prepared in phosphate buffer, pH 7.4. The EIS data of the Nyquist plot are shown in Figure 6 B.
